# Automated partial atomic charge assignment for drug-like molecules: a fast knapsack approach

**DOI:** 10.1186/s13015-019-0138-7

**Published:** 2019-02-05

**Authors:** Martin S. Engler, Bertrand Caron, Lourens Veen, Daan P. Geerke, Alan E. Mark, Gunnar W. Klau

**Affiliations:** 1 Algorithmic Bioinformatics, Heinrich Heine University, Universitätsstr. 1, 40225 Düsseldorf, Germany; 2 Life Sciences and Health Group, Centrum Wiskunde & Informatica, Science Park 123, 1098 XG Amsterdam, The Netherlands; 3 School of Chemistry & Molecular Biosciences, The University of Queensland, Brisbane, QLD 4072 Australia; 4 Netherlands eScience Center, Science Park 140, 1098 XG Amsterdam, The Netherlands; 5 AIMMS Division of Molecular and Computational Toxicology, Vrije Universiteit, De Boelelaan 1108, 1081 HZ Amsterdam, The Netherlands

**Keywords:** Multiple-choice knapsack, Integer Linear Programming, Pseudo-polynomial Dynamic Programming, Partial charge assignment, Molecular dynamics simulations

## Abstract

A key factor in computational drug design is the consistency and reliability with which intermolecular interactions between a wide variety of molecules can be described. Here we present a procedure to efficiently, reliably and automatically assign partial atomic charges to atoms based on known distributions. We formally introduce the molecular charge assignment problem, where the task is to select a charge from a set of candidate charges for every atom of a given query molecule. Charges are accompanied by a score that depends on their observed frequency in similar neighbourhoods (chemical environments) in a database of previously parameterised molecules. The aim is to assign the charges such that the total charge equals a known target charge within a margin of error while maximizing the sum of the charge scores. We show that the problem is a variant of the well-studied multiple-choice knapsack problem and thus weakly $\mathcal {NP}$-complete. We propose solutions based on Integer Linear Programming and a pseudo-polynomial time Dynamic Programming algorithm. We demonstrate that the results obtained for novel molecules not included in the database are comparable to the ones obtained performing explicit charge calculations while decreasing the time to determine partial charges for a molecule from hours or even days to below a second. Our software is openly available.

## Introduction

Molecule-based computational modelling and simulation studies play a central role in modern drug design and development. In particular, molecular dynamics (MD) simulations and free energy calculations are increasingly being used to screen potential ligand molecules in terms of their interactions with proposed target molecules (e.g. cell surface receptors or enzymes involved in metabolism) [1, 2]. They are also used to model structural changes in the target molecule associated with the binding of a given drug in order to understand the mechanism of action. The accuracy and utility of such modelling studies depends directly on the fidelity with which intermolecular interactions can be represented [3, 4]. While ideally one might wish to represent such interactions on the level of quantum mechanics, the size and complexity of protein-ligand complexes necessitates the use of classical dynamics in conjunction with empirical potentials. These so-called force fields are parameterised to reproduce the interactions between atoms in a system of interest (e.g. protein, membrane, drug) and involve bonds, angles, dihedrals, van der Waals and Coulomb interactions.

Of particular importance is the assignment of partial atomic charges to describe the latter interactions. Partial atomic or point charges are used to represent the electrostatic potential around a molecule and the Coulomb interactions between these point charges dominate the calculation of intermolecular interactions. The difficulty is that the effective partial charge on an atom needed to represent the electrostatic potential surrounding a molecule is heavily dependent on the local environment in which an atom is found. For small molecules (< 40 atoms) partial atomic charges can generally be inferred *de novo* from quantum-mechanical computations [5]. However, when using e.g. commonly applied Density Functional Theory (DFT) such calculations scale cubic in the number of valence electrons [6], increasing the computational costs significantly. In addition, as molecules become larger the accuracy with which charges can be assigned decreases.

A standard approach to address this problem is to manually assign charges to atoms based on their similarity to atoms (or groups) in a set of reference molecules containing equivalent chemical moieties. The challenge in making such assignments is twofold: (1) the charges assigned to equivalent chemical groups in alternative reference molecules may vary, making the choice of a reference molecule difficult and (2) the charges assigned to neighbouring atoms must be consistent. In particular, the total charge on the molecule must be integer. In recent years, a number of machine learning approaches emerged that infer charges based on a set of reference molecules [7–9]. However, these approaches often struggle to deal with the ambiguity of similar groups that have different charges in different molecules and the requirement that the overall charge must be integer.

In this paper, we consider the problem of—given a large set of reference molecules with known charge distributions—how to efficiently, automatically and optimally assign partial atomic charges which are consistent with both the neighbouring atoms and the total charge. As a reference we have used molecules parameterised using the Automated Topology Builder (ATB) and repository [3]. The ATB contains a large number of molecules (< 50 atoms) for which partial charges have been assigned *de novo*. In previous work, we have contributed to improving the reliability of this repository by ensuring the consistency and utility of the partial charges assigned to atoms by identifying atoms that could be used to form *charge groups*, which can be collectively assigned integer formal charges ($\ldots ,-1,0,1,\ldots$) [10]. We have also developed methods to match molecular substructures, taking into account that the partial charge of an atom is heavily dependent on its neighbours and the nature of its local chemical environment [11]. This made it possible to study the distribution of charges within local molecular environments for all molecules in the ATB ($\approx$ 260,000 molecules; 9,000,000 atoms and 9,100,000 bonds) and to find, given a query molecule, all possible matching fragments (sub-graphs).

Here we build on this previous work and our ability to match sub-fragments of a query molecule against the available database, to consider how the information contained in already parameterised molecules can be used best to infer the charges within a novel molecule. The most direct approach would be to assign a simple average partial charge on individual atoms identified as equivalent using a given similarity criterion. However, quantum mechanics dictates that the total charge on a molecule must be integer. Simply attributing to each atom the value of an average partial charge from the known distribution fails as it results in the accumulation of errors and a total charge deviating from the required value.

Instead, we have considered solutions to the *molecular charge assignment problem*, which allow charges that deviate from the average to be selected while their sum is constrained to lie close to a target total charge. Among the possible set of solutions we prefer those that maximize a score that depends on the observed frequencies of the chosen charges. We show that the problem is similar to a *multiple-choice knapsack problem* (MCKP) [12, 13]. We introduce $\epsilon$-MCKP, a variant of the standard MCKP with an error margin $\epsilon$. We provide an Integer Linear Programming (ILP) formulation of $\epsilon$-MCKP and adapt the MCKP pseudo-polynomial Dynamic Programming (DP) algorithm to $\epsilon$-MCKP. By evaluating the difference of the charges assigned by solving the $\epsilon$-MCKP approach for the charge assignment problem to the *de novo* computed ATB charges, we find that they are comparable while decreasing the time to determine partial charges for a molecule by several orders of magnitude, that is, from hours or even days for the latter to below a second for the $\epsilon$-MCKP solution. Finally, we show that our automated method yields similar results on a large example molecule as an expert-supervised semi-automatic approach [14].

Our code is publicly available under the Apache 2.0 open source license [15].

## Assigning charges

We consider molecules as graphs. Let $G = (V, E, t)$ be a *molecular graph*, where vertices *V* correspond to atoms, edges *E* correspond to bonds and $t: V \rightarrow \Sigma$ colors vertices with atom types. A straightforward alphabet of atom types $\Sigma$ would be the chemical elements. In this work, we used ATB-assigned GROMOS atom types which provide a more detailed classification of some chemical elements (N, C, O, S) depending on their hybridization (number of bonded nodes), and therefore provides a more detailed description of the local environment.

The partial charge of an atom is heavily dependent on its bonded neighbours and the nature of its local environment. Formally, we define the *k*-neighbourhood as:

### **Definition 1**

(*k-neighbourhood*) Let $N(v) = \{u \mid (u, v) \in E\}$ be the neighbourhood of an atom *v*. We define the *k-neighbourhood* recursively as $N_k(v) = N_{k-1}(v) \cup \bigcup _{u \in N_{k-1}(v)} N(u)$, with $N_0(v) = {v}$.

Informally, the *k*-neighbourhood of an atom *v* is the set of all atoms for which a path of length $\le k$ to *v* exists. Let $G[N_k(v)]$ be the subgraph induced by the *k*-neighbourhood of *v*.

To collect all possible partial charge values, we consider all *k*-neighbourhoods in the set of previously parameterised molecules. For this we iterate over all atoms *v* of all molecular graphs in the ATB and construct a list of subgraphs $G[N_k(v)]$ with associated partial charges of the corresponding atom *v*. We construct a database with an entry for each isomorphism class in the subgraph list. For each isomorphism class we collect the partial charges of its subgraphs and condense the values to a histogram. Since the point charges assigned by the ATB are rounded to three digits after the decimal point, we round the partial charge values accordingly.

**Fig. 1:**
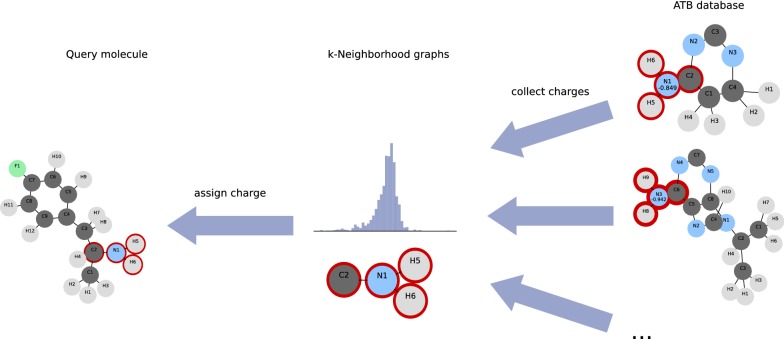
General overview. Given a query molecule, our method assigns atomic partial charges based on matching isomorphic subgraphs (red) with a known partial charge distribution collected from the ATB database of parameterised molecules

Given a query molecule with a known target total charge, the challenge is to assign the most representative partial charge to each atom while staying close to the target total charge (Fig. 1). For that purpose, we iterate over all atoms of the query molecular graph and generate the subgraphs $G[N_k(v)]$. We match each subgraph to its isomorphism class in our database of *k*-neighbourhood subgraphs. If there is no match, we iteratively retry with $G[N_{k-1}(v)]$ until $k = 0$. Now each atom in our query molecule has a histogram of possible partial charges. The task is now to assign the charges such that we maximize the frequencies of the assigned charges while the sum of assigned partial charges equals the target charge with some error margin.

## Problem formulation and complexity

We map each atom *i* to a set of items *j* with weights $w_{i,j}$ corresponding to partial charges and profits $p_{i,j}$ corresponding to their frequency-based scores. The target total charge corresponds to capacity *c*. Note that the charge assignment problem is now similar to a multiple-choice knapsack problem (MCKP). The decision version of MCKP is defined as:

### **Problem 1**

(MCKP) Given a decision variable $K \ge 0$, capacity $c \ge 0$, *m* sets $N_1, \dots , N_m$ of items $j \in N_i$ with profit $p_{i,j} \ge 0$ and weight $w_{i,j} \ge 0$, select exactly one item from each set, such that the sum of weights of the selected items does not exceed *c* and the sum of profits of the selected items is equal or larger than *K*.

MCKP is known to be weakly $\mathcal {NP}$-complete [12, 13, 16]. However, although the problem of assigning charges is similar to MCKP, there are two differences. First, weights and capacity can be negative numbers. Second, the sum of weights of selected items must hit the capacity with some error margin, resulting in an upper and lower capacity limit. We define a variant of MCKP, which is equivalent to the charge assignment problem as:

### **Problem 2**

($\epsilon$*-MCKP*) Given a decision variable $K \ge 0$, capacity $-\infty \le c \le \infty$, error $\epsilon \ge 0$, *m* sets $N_1, \dots , N_m$ of items $j \in N_i$ with profit $p_{i,j} \ge 0$ and weight $-\infty \le w_{i,j} \le \infty$, select exactly one item from each set, such that the sum of weights of the selected items is in the range $[c - \epsilon , c + \epsilon ]$ and the sum of profits of the selected items is equal or larger than *K*.

### **Theorem 1**

$\epsilon$*-MCKP is weakly* $\mathcal {NP}$*-complete*.

### *Proof*

Showing that $\epsilon$-MCKP is in $\mathcal {NP}$ is straightforward. Given an instance of $\epsilon$-MCKP and a candidate solution $\hat{S}$, we can easily check in polynomial time whether $c-\epsilon \le \sum _{w_{i,j} \in {\hat{S}}} w_{i,j} \le c + \epsilon$ and $\sum _{p_{i,j} \in \hat{S}} p_{i,j} \ge K$ as well as if $\hat{S}$ contains exactly one item from each set $N_1, \dots , N_m$. We show that $\epsilon$-MCKP is weakly $\mathcal {NP}$-hard as follows: We reduce MCKP $\le _\textsc {p}$ $\epsilon$-MCKP. Given an instance of the standard MCKP with capacity *c*, we transform it to an $\epsilon$-MCKP instance with capacity $c' = \frac{1}{2} c$ and $\epsilon = \frac{1}{2} c$. Then, $c' - \epsilon = 0$ and $c' + \epsilon = c$, making both instances equivalent. $\square$

Both problems obviously can be transformed into optimization problems by omitting the decision variable *K* and maximizing the sum of profits. The definition of $\epsilon$-MCKP allows us to solve the charge assignment problem.

## Solving $\epsilon$-MCKP

In this section we present two algorithmic strategies to solve $\epsilon$-MCKP: the first is based on an Integer Linear Programming (ILP) formulation, which can be solved by general ILP solvers, while the second is a purely combinatorial Dynamic Programming (DP) algorithm.

Formulating $\epsilon$-MCKP as an ILP is straightforward. Let $x_{i,j}$ be a binary variable with value 1 if and only if item *j* in set $N_i$ is selected. We formulate the problem as:

1a $$\begin{aligned}&\max \quad \sum \limits _{i = 1}^{m} \sum \limits _{j \in N_i} x_{i,j} p_{i,j} \end{aligned}$$

1b $$\begin{aligned}&\text {subject to} \quad \sum \limits _{i = 1}^{m} \sum \limits _{j \in N_i} x_{i,j} w_{i,j} \ge c - \epsilon \end{aligned}$$

1c $$\begin{aligned}&\sum \limits _{i = 1}^{m} \sum \limits _{j \in N_i} x_{i,j} w_{i,j} \le c + \epsilon \end{aligned}$$

1d $$\begin{aligned}&\sum \limits _{j \in N_i} x_{i,j} = 1 \quad \text {for} \, 1 \le i \le m \end{aligned}$$

1e $$\begin{aligned}&x_{i,j} \in \{0, 1\} \quad \text {for} \, 1 \le i \le m, j \in N_i \end{aligned}$$

The second algorithm is an adaption of the pseudo-polynomial DP of the standard MCKP to $\epsilon$-MCKP. The standard MCKP assumes numbers to be non-negative integers. If a given $\epsilon$-MCKP instance does not comply with the non-negativity and integrality constraints, we transform the instance as follows:

First, we convert floating point weights $w_{i,j}$, capacity *c* and error $\epsilon$ to integers by multiplying with an appropriate factor. Since point charges in this work are rounded to three digits after the decimal point, a factor of $10^3$ is sufficient. Second, we transform the weights $w_{i,j}$ and capacity *c* to non-negative numbers. For every set $N_j$ with $j = 1, \dots , m$, we determine the minimum weight $w^*_i = \min _{j \in N_i} w_{i, j}$. We define the new weights as $\tilde{w}_{i,j} = w_{i,j} - w^*_i$. Then, the weights are guaranteed to be non-negative. As we have to select one item per set, we can define the new capacity as $\tilde{c} = c - \sum _{i = 1}^{m} w^*_i$.

Therefore, we assume in the following (without loss of generality) that weights $w_{i,j}$, capacity *c* and error $\epsilon$ are non-negative integers. Let *P* be a two-dimensional DP-table of size $m \times (c + \epsilon )$. *P*[*k*, *d*] holds the maximum profit that we can achieve with sets 0 to *k* and a sum of weights of exactly *d*:

2 $$\begin{aligned} P[k,d] = \max \left\{ \sum _{i = 0}^{k} \sum _{j \in N_i} x_{i, j} p_{i, j} : \sum _{i = 0}^{k} \sum _{j \in N_i} x_{i, j} w_{i, j} = d , \sum _{j \in N_k} x_{i, j} = 1 \, \text {for all} \, 0 \le i \le k \right\} \end{aligned}$$

We compute *P* recursively. Let *P*[*k*, *d*] be defined as:

3 $$\begin{aligned} P[k,d] = \max {\left\{ \begin{array}{ll} P[k - 1, d - w_{k, j}] + p_{k, j} &{} \text {for } j \in N_i \text { and } d - w_{k, j} \ge 0 \\ -\infty &{} \end{array}\right. } \end{aligned}$$

*P*[*k*, *d*] is calculated by considering all items of the current set $N_k$ and computing the maximum profit that can be achieved when adding those profits to possible previous solutions with $k - 1$ sets and sum of weights $d - w_{k, j}$. The profit is $-\infty$ if there is no possible solution for *P*[*k*, *d*]. Contrary to the standard MCKP DP we initialize *P* as:

4 $$\begin{aligned} P[0,d] = {\left\{ \begin{array}{ll} 0 &{} \text {if } d = 0 \\ -\infty &{} \text {otherwise} \end{array}\right. } \end{aligned}$$

This ensures that only solutions in which the sum of selected weights equals exactly *d* are possible. We find the maximum profit $p^*$ by:

5 $$\begin{aligned} p^* = \max \left\{ P[m, d]: \max \{c - \epsilon , 0\} \le d \le c + \epsilon \right\} \end{aligned}$$

The DP can be easily implemented using one dimension, as the recursion only looks back one step in the dimension *k* (the number of sets we currently consider). The space requirement of the DP algorithm is $O(c + \epsilon )$. The running time complexity is $O(n(c + \epsilon ))$, with *n* being the total number of items.

## Results and discussion

To evaluate our method, we conducted a leave-one-out-analysis using a snapshot of the ATB database containing roughly 145,000 molecules with charges assigned based on electrostatic-potential fitting using quantum mechanical calculations at theory level 1 and 2 [3]. We focus on this set of previously computed molecules, since the computational effort of large-scale quantum-mechanical calculations is significant. We created a database of *k*-neighbourhood subgraphs associated with partial charge histograms with variable bin widths and a fixed $k = 3$. Bin widths were determined according to the Friedman–Diaconis rule [17] and bin centers were aligned to the median charge.

**Fig. 2:**
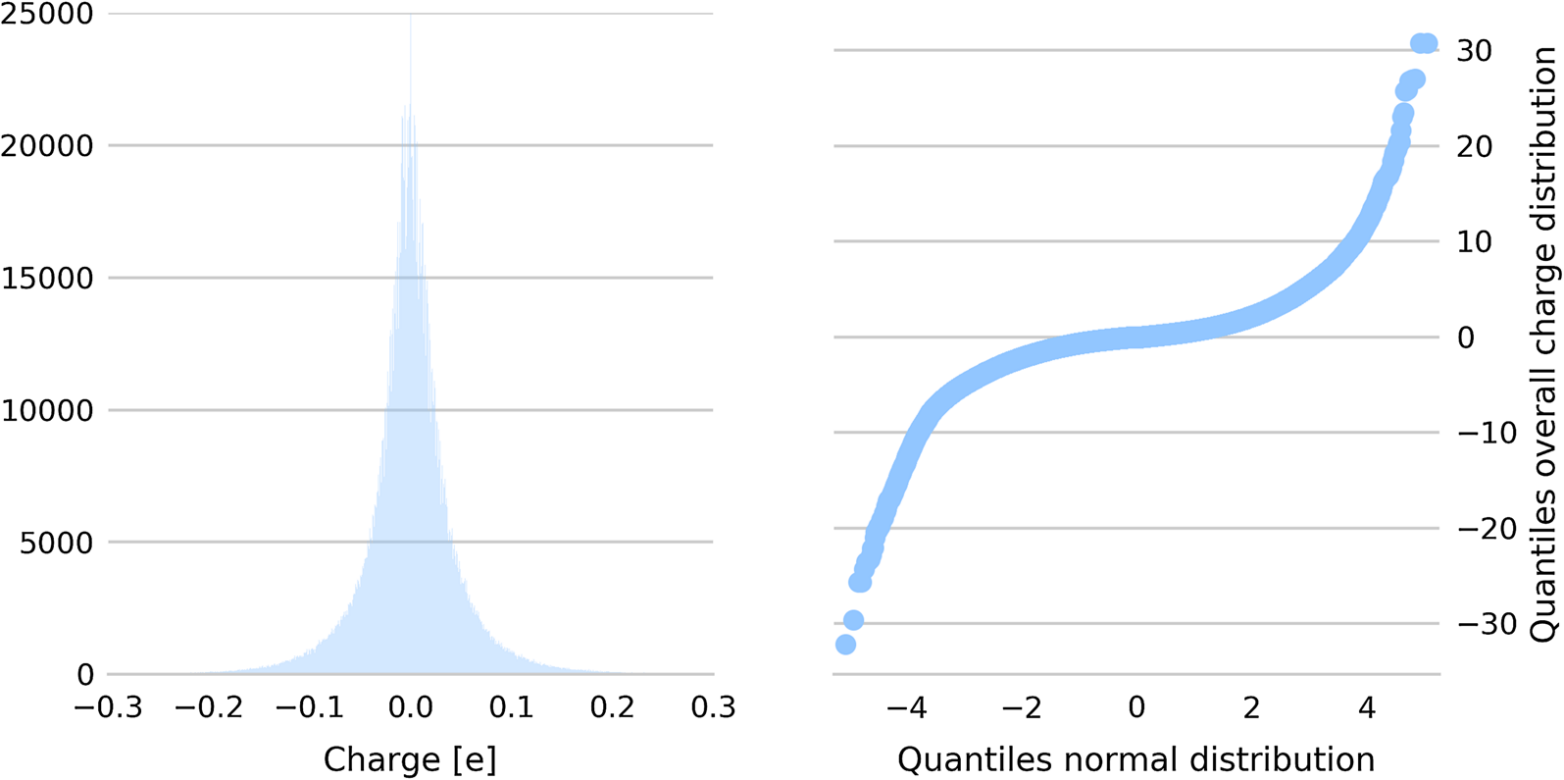
Charge distribution over all 3-neighborhood graphs. Distribution of charges over all 3-neighbourhood graphs centered at the sample mean of each 3-neighbourhood graph (left) and Q–Q-plot with the quantiles of the charge distribution over all 3-neighbourhood graphs on the y-axis and the quantiles of a fitted normal distribution on the x-axis

Figure 2 shows the distribution of charges over all 3-neighbourhood graphs in the snapshot of the ATB that are centered at the sample mean of each 3-neighbourhood graph. Evidently, the distribution is heavy-tailed and not Gaussian. Therefore, as profits $p_{i,j}$ for the $\epsilon$-MCKP problem we decided in favor of the log of the bin counts instead of scoring functions which assume normally distributed data such as the z-score.

For each molecule in the snapshot, we filtered out all charge values associated with its 3-neighbourhood subgraphs for each molecule and computed the atomic partial charges using the remaining data. For the 67,572 molecules that could be fully covered with a fixed shell size of $k = 3$ (that is, for each atom a matching 3-neighborhood was found), we compared the assigned values to the original atomic partial charges in the ATB database.

**Fig. 3:**
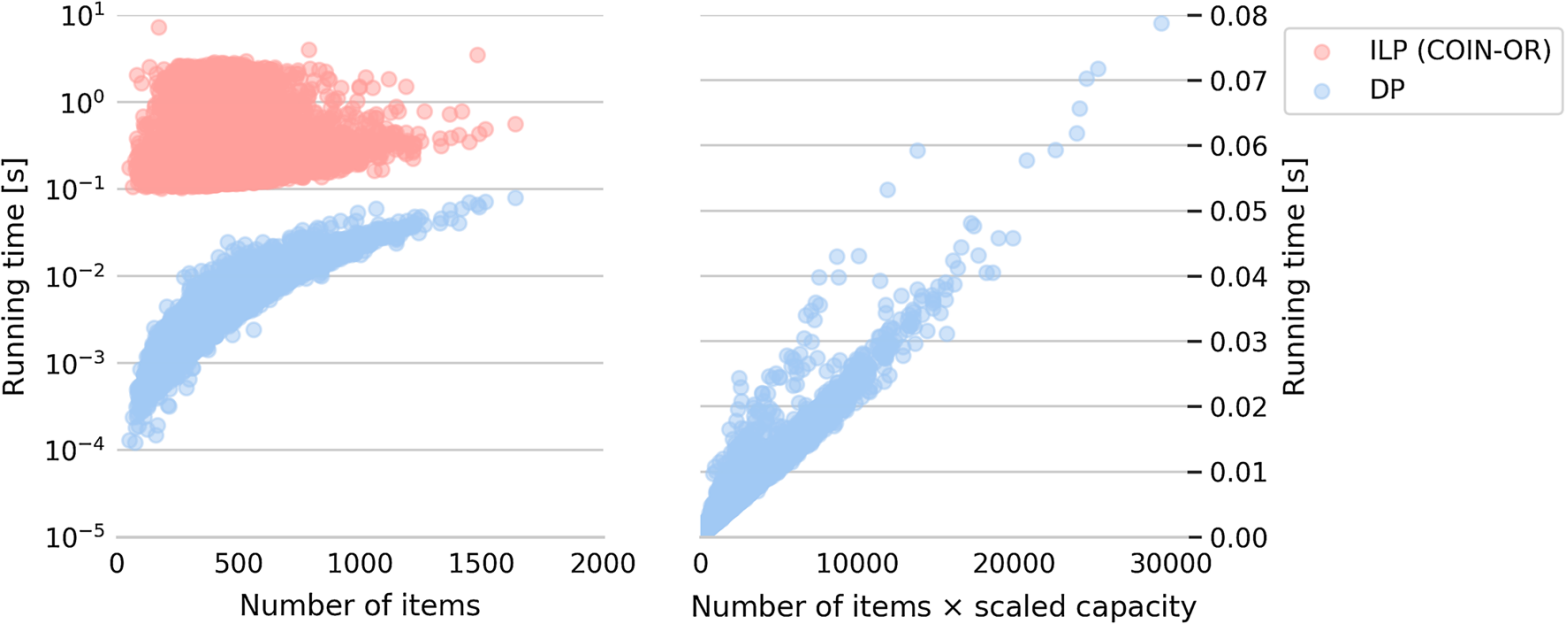
Running times. Running times of the ILP solved with COIN-OR and the DP dependent on the number of items *n*, showing that the DP is significantly faster than the ILP (left). The running time of the DP actually depends on the number of items times the scaled capacity $10^3 \cdot n \cdot (\tilde{c} + \epsilon )$ (right). Note that one instance is not shown for visibility reasons. The excluded instance has 3640 items and running times of 0.42 s and 1.26 s for the DP and ILP, respectively

All computations were performed on a compute cluster with 16 cores at 3.2 GHz and 64 GB RAM per node. The ILP was solved using COIN-OR [18]. We recorded the running times of the ILP and DP algorithm, see Fig. 3. As expected, the running time of the DP scales linearly with $10^3 \cdot n \cdot (\tilde{c} + \epsilon )$, where *n* is the number of items, $10^3$ is the blowup factor and $\tilde{c}$ is the capacity *c* transformed to non-negativity with $\tilde{c} = c - \sum _{i = 1}^{m} w^*_i$ and $w^*_i = \min _{j \in N_i} w_{i, j}$. The running times of the ILP show more variation and a marginal positive correlation to the number of items *n*, which equals the number of variables in the ILP. The DP was always significantly faster than the ILP in the leave-one-out-evaluation. It should be noted however, that both methods are orders of magnitude faster than the *de novo* electrostatic-potential based charge assignment using quantum-mechanical computations. For example, for the molecule ATB ID 25338 with 120 atoms, the *de novo* method required $\sim 140$ days while solving our $\epsilon$-MCKP approach was finished in $\sim 0.12$ (ILP) and $\sim 0.06$ (DP) seconds with both methods using a single core.

**Fig. 4:**
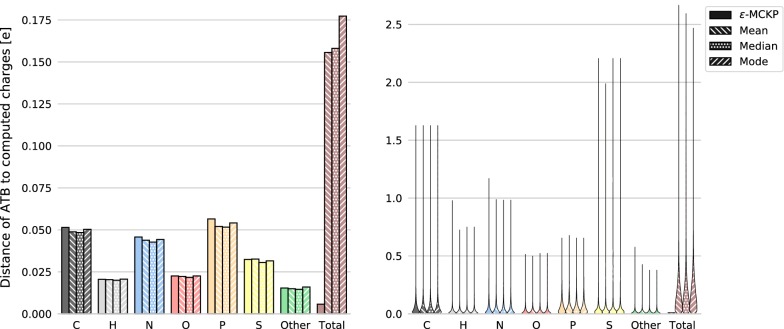
Accuracy evaluation. Results of the leave-one-out experiment with *k* = 3 showing mean distances in elementary charge units (left) and violin plots of all distances (right) of original charges found in the ATB to charges calculated by different methods: solving $\epsilon$-MCKP, and selecting the mean, median, and mode of the possible charge values for each atom. For $\epsilon$-MCKP, the maximal allowed difference $\epsilon$ to the target total molecular charge was set to 0.01. The distances are categorized by chemical element, and given for the total molecular charge as well (Total)

In the leave-one-out evaluation, we compared the naive approaches of estimating the atomic partial charges by simply choosing the mean, median or mode of the charge distributions per atom, with our method of solving $\epsilon$-MCKP instances, see Fig. 4. In case of multimodal charge distributions, we selected the mode closest to the median. As expected, while the naive methods are, on average, able to find charges with a slightly lower distance to the original partial charges, their use often results in a total molecular charge far away from the target total charge (with errors of more than 1*e* in many cases). Our method on the other hand is able to assign partial charge values which are similar to the ones computed by the naive methods while achieving a total molecular charge close to the target total charge (with a maximal allowed difference of $\epsilon = 0.01e$).

**Fig. 5:**
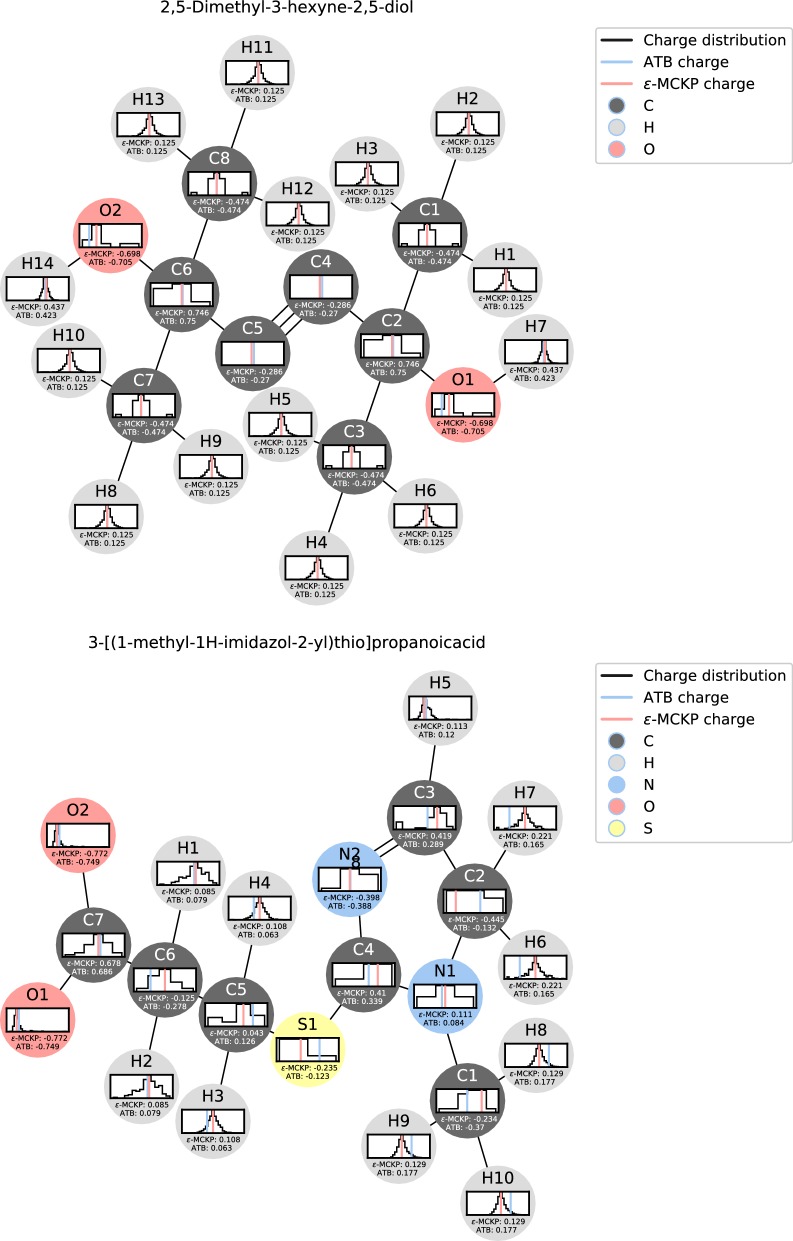
Example molecules. Molecules with closest (top) and furthest (bottom) average distance between ATB and $\epsilon$-MCKF determined charges in the leave-one-out evaluation. Atoms (nodes) are color-coded by their chemical element (red for oxygen, blue for nitrogen, black for carbon, yellow for sulfur and grey for hydrogen). Atoms are overlaid with the histograms of the leave-one-out charges of their respective 3-neighbourhoods. The original ATB charge and the computed charges are shown by blue and red vertical lines in the histograms

As an example of the charges assigned by our method, Fig. 5 shows the two molecules with the atomic partial charges that are on average closest to and farthest from the original ATB charges. The computed charges for the molecule with the closest distance fit well to the original ATB charges. $\epsilon$-MCKP assigns identical charges to atoms H1, H2 and H3. The 3-neighbourhood graphs of all three atoms have the same isomorphism class. This is an advantage of our $\epsilon$-MCKP approach, since quantum-mechanical *de novo* charge assignment does not guarantee that similar charges are assigned to equivalent atoms (although in this case the ATB charges are also identical). For the molecule with the farthest distance the largest individual distance is 0.31*e*. We observe that the large distances are often caused by the original ATB charges being on the outer edges of the charge distributions, while $\epsilon$-MCKP on the other hand most of the time picks charges close to the largest mode of the distribution, see bottom side of Fig. 5. Note that Fig. 5 shows the distributions used in the leave-one-out evaluation without the original ATB charges of the depicted molecule. Additionally, the charge distributions of atoms with large charge distances have been computed with a low number of observed charges, resulting in multimodal distributions with several large peaks. We expect this effect to disappear when more data is available in the constantly growing ATB repository.

Most of the outer atoms—especially the hydrogens—showed a narrow unimodal distribution of ATB charges and $\epsilon$-MCKP picked charges close to the original ATB charge. The more buried atoms showed a larger variability. For some buried atoms, we observed a similar behavior as for e.g. S1, C5, and C6 in Fig. 5, that $\epsilon$-MCKP selects a charge closer to the distribution mean than the original ATB charge was. However, for several atoms we observed the limit of our data-driven approach. If only a few charge values are available for a certain *k*-neighbourhood, then the distributions are multimodal with very similar or equal peak heights, reflecting the variability of the quantum-mechanically derived charges. Then, $\epsilon$-MCKP may freely choose between co-optimal solutions. On the other hand, if only exactly one charge value is available, $\epsilon$-MCKP has to choose this value. While the probability of this occurring will decrease with the addition of more data, in this case (with the current dataset) it could be advisable to use a smaller neighborhood size *k*. With choosing an appropriate *k*, the user may balance the specificity of large *k*-neighbourhoods against the robustness of small *k*-neighbourhoods.

In general, charges on the outer atoms of a molecule can be assigned quite well while charges of the inner atoms deviate more from the ATB charges. This may be explained by the larger variability of the inner atoms in the ATB dataset, an artifact of the *de novo* electrostatic-potential based charge assignment [19].

**Fig. 6:**
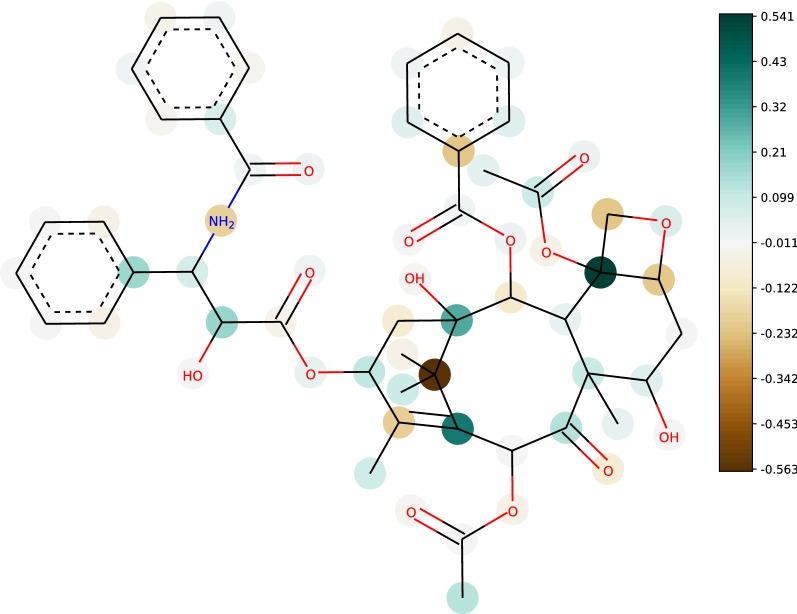
Large example molecule. Comparison of $\epsilon$-MCKP to expert assignments using Paclitaxel with 113 atoms. Atoms are marked according to the distance (in elementary charge units) of the mean of five expert-chosen partial charges to the partial charges computed by $\epsilon$-MCKP. Note that aliphatic hydrogens are not shown and their charge distances have been added to their respective neighbours

Additionally, we compared $\epsilon$-MCKP to the expert-supervised semi-automated method OFraMP [14]. For a query molecule, OFraMP visually suggests maximal common substructures matching molecules in the ATB database. This allows an expert to construct a molecular parameterisation by selecting the appropriate partial charges from the substructures and redistributing the distance of the sum of assigned partial charges within the query to the target total molecular charge. For the anti-cancer drug Paclitaxel (with 113 atoms), we compared our $\epsilon$-MCKP solution to the mean partial atomic charges from five different assignments obtained by an expert using OFraMP [14], see Fig. 6. To find the matching *k*-neighborhoods we iterated over shell size *k* from 3 to 1 for each atom until an isomorphic *k*-neighborhood was found.

Overall, $\epsilon$-MCKP and the expert assignments agree quite well with absolute distances less than 0.01*e* for 28 atoms and less than 0.1*e* for 98 atoms. However, two buried carbon atoms show absolute distances of approximately 0.5*e*. We identified two possible causes. First, the differences may be explained by the larger variability of the partial charges of buried atoms in the ATB dataset, which is in line with Fig. 6 where atoms in the center of the molecule show the highest differences. For example, one of the two buried carbon atoms in question already shows a high variability in the five expert-supervised assignments with a standard deviation of 0.17*e*. Second, we observed a correlation of the distances to the number of molecules in the ATB where the *k*-neighborhoods were found and how specific the *k*-neighborhood is, i.e. how large the shell size *k* is. The fewer ATB molecules support a *k*-neighborhood and the smaller its shell size is, the larger is the difference between our method and the expert assignments. This confirms that our method may well perform even better when using a larger dataset of molecules.

## Conclusions

The ability to accurately calculate the electrostatic interactions between a ligand and its receptor is a key component of computer-aided drug development. In this paper, we have investigated the problem of automatically assigning partial charges. The charge assignment problem is similar to the multiple-choice knapsack problem. We introduced a variant tailored to the charge assignment problem, the $\epsilon$-multiple-choice knapsack problem ($\epsilon$-MCKP). Like most knapsack problems, $\epsilon$-MCKP is weakly $\mathcal {NP}$-complete. We presented two algorithmic solutions to $\epsilon$-MCKP, an Integer Linear Programming (ILP) formulation and a Dynamic Programming (DP) algorithm.

We conducted a leave-one-out evaluation on a snapshot of the ATB database. The computed atomic partial charges were close to the original ATB charges and the total charge virtually the same as the target total charge. This suggests that our method provides consistent parameters for MD simulations, docking studies and other related applications. One additional advantage of our approach is that equivalent nodes in the graph will be assigned similar charges and the charge distribution will therefore mirror the symmetry of the molecular graph. The additional comparison to expert-assigned charges for a large and complex drug molecule (Paclitaxel) supports these conclusions, but it also showed that the quality of the computed charges correlates to the size of the database used by our method. As the ATB is constantly growing, we are confident that our method may also constantly improve.

The DP algorithm performed faster than the ILP on a set of 160,000 molecules contained within the ATB. On average, both implementations required only a fraction of a second to assign charges to molecules containing 50–100 atoms, while quantum-mechanical computations required many days. This is important when screening large molecular databases. For instance, ChEMBL [20], a manually curated chemical database of bioactive molecules with drug-like properties, contains in excess of 1.6 million compounds. The majority of these have more than 50 atoms making quantum-mechanical computations difficult. Other computational drug design databases are larger again [21]. For example, ZINC, a free database of commercially-available compounds, contains more than 35 million compounds [22].

Our method builds on a repository of previously computed molecular parameters and assigns consistent partial atomic charges in a swift manner to facilitate MD simulations and related applications in drug design.
